# In Vitro 3D Spheroid Culture System Displays Sustained T Cell-dependent CLL Proliferation and Survival

**DOI:** 10.1097/HS9.0000000000000938

**Published:** 2023-08-23

**Authors:** Marco V. Haselager, Bianca F. van Driel, Eduard Perelaer, Dennis de Rooij, Danial Lashgari, Remco Loos, Arnon P. Kater, Perry D. Moerland, Eric Eldering

**Affiliations:** 1 Department of Experimental Immunology, Amsterdam UMC Location University of Amsterdam, Meibergdreef, The Netherlands; 2 Lymphoma and Myeloma Center Amsterdam, LYMMCARE, Amsterdam, The Netherlands; 3 Cancer Center Amsterdam, Amsterdam Institute for Infection and Immunity, Amsterdam, The Netherlands; 4 Amsterdam Institute for Infection and Immunity, Cancer Immunology, Amsterdam, The Netherlands; 5 Department of Hematology, Amsterdam UMC Location University of Amsterdam, Meibergdreef, The Netherlands; 6 Department of Epidemiology and Data Science, Amsterdam UMC Location University of Amsterdam, Meibergdreef, The Netherlands; 7 Center for Innovation and Translational Research Europe, Bristol Myers Squibb, Sevilla, Spain; 8 Amsterdam Institute for Infection and Immunity, Inflammatory Diseases, Amsterdam, The Netherlands; 9 Amsterdam Public Health, Methodology Amsterdam, The Netherlands

## Abstract

Chronic lymphocytic leukemia (CLL) cells are highly dependent on microenvironmental cells and signals. The lymph node (LN) is the critical site of in vivo CLL proliferation and development of resistance to both chemotherapy and targeted agents. We present a new model that incorporates key aspects of the CLL LN, which enables investigation of CLL cells in the context of a protective niche. We describe a three-dimensional (3D) in vitro culture system using ultra-low attachment plates to create spheroids of CLL cells derived from peripheral blood. Starting from CLL:T cell ratios as observed in LN samples, CLL activation was induced by either direct stimulation and/or indirectly via T cells. Compared with two-dimensional cultures, 3D cultures promoted CLL proliferation in a T cell-dependent manner, and enabled expansion for up to 7 weeks, including the formation of follicle-like structures after several weeks of culture. This model enables high-throughput drug screening, of which we describe response to Btk inhibition, venetoclax resistance, and T cell-mediated cytotoxicity as examples. In summary, we present the first LN-mimicking in vitro 3D culture for primary CLL, which enables readouts such as real-time drug screens, kinetic growth assays, and spatial localization. This is the first in vitro CLL system that allows testing of response and resistance to venetoclax and Bruton's tyrosine kinase inhibitors in the context of the tumor microenvironment, thereby opening up new possibilities for clinically useful applications.

## Graphical abstract

**Figure:**
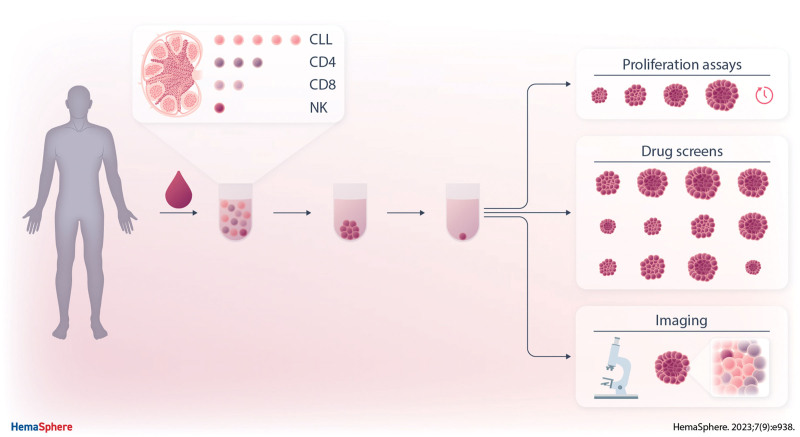

## INTRODUCTION

Chronic lymphocytic leukemia (CLL) is a prime example of a malignancy that is highly dependent on interactions with the microenvironment. At lymph node (LN) sites, CLL cells are provided with external signals from surrounding cells such as CD40L-presenting T helper cells, myeloid, and stromal cells, creating a protective niche that is thought to play a key role in the long-term response to targeted agents and development of resistance.^1^ Currently, CLL biology and response to drugs are widely studied using primary leukemia cells isolated from peripheral blood (PB), which has important drawbacks given the fact that in vivo CLL growth occurs at LN sites.^2^ Although coculture with stromal cells or the addition of soluble factors can increase and extend survival of CLL cells, no existing systems permit the long-term expansion of CLL cells in vitro.^3^ Moreover, current models cannot be used to study adhesion, migration, or immune responses in the context of the tumor microenvironment. Despite similarities between human and murine models for CLL, inherent differences pose important limitations, such as the lack of LN involvement in most commonly used CLL mouse models.^4^

Development of three-dimensional (3D) models that are able to accurately mimic the CLL microenvironment may help to bridge the gap between current in vitro systems and the physiologic CLL microenvironment in vivo, overcoming some of the current limitations of in vitro CLL studies. Recent efforts have been made toward the development of bone marrow-specific CLL models,^5,6^ yet it is currently accepted that the LN is the critical site of CLL proliferation.^2^ We attempted to establish a model that incorporates key aspects of CLL LN biology, taking into account physiological relevance. We formulated several criteria that the system should meet: contribution of non-CLL cells, long-term proliferation, induction of drug resistance, high-throughput, and practical to use.

Here, we present a novel 3D LN model for the in vitro culture of CLL that mimics a LN microenvironment using PB samples of CLL patients. By mimicking the cellular composition of the LN in combination with the addition of soluble factors to resemble the extracellular microenvironment, in this way, we created a model where multiple upstream signals activate divergent downstream signaling pathways in CLL cells, resulting in phenotypes that allow for better investigation of CLL proliferation and high-throughput drug screening. In this article, we demonstrate the principle of the model including several functional readouts such as proliferation, spatial localization, and drug resistance. We provide the protocols and details in order for other research groups to adopt this system. Moreover, this model can be adapted and used for the generation of more complex tumor microenvironments tailored to specific research objectives.

## MATERIALS AND METHODS

### Patient material

With written informed consent of the patients, the blood samples were obtained during diagnostic or follow-up procedures at the Departments of Hematology and Pathology of the Academic Medical Center (AMC), Amsterdam. This study was approved by the AMC Ethical Review Biobank Board under the number METC 2013/159 and conducted in accordance with the Declaration of Helsinki. Blood mononuclear cells of patients with CLL, obtained after Ficoll density gradient centrifugation (Pharmacia Biotech, Roosendaal, The Netherlands), were cryopreserved and stored as previously described.^7^ Cells were stained with the following antibodies: CD8, CD56, CD5, CD19, TCRγδ, CD3, CD4, and CD20. Cells were measured by flow cytometry on a Canto II flow cytometer (BD Biosciences, Franklin Lakes, New Jersey [NJ]). We used CLL samples containing 50%–90% CD5^+^/CD19^+^ CLL cells and >10% CD4^+^ T cells. Paired PB and LN samples were characterized in the context of the HOVON 158 clinical trial. All patient samples included in this study are listed in Suppl. Table S1.

### Protocols

All used protocols are listed in the Supplemental Information together with corresponding reagents and equipment.

### Cell culture

Peripheral blood mononuclear cells (PBMCs) of CLL patients were plated in standard round-bottom 96-well culture plates (two-dimensional [2D]) or in ultra-low attachment (ULA) plates and centrifuged for 10 minutes at 1000 rpm and subsequently incubated for 24 hours to allow spheroid formation (3D). Cells were cultured at 37°C and 5% CO_2_ using standard cell culture techniques. Cells were cultured in Iscove’s Modified Dulbecco’s Medium supplemented with 10% fetal calf serum and 1% penicillin/streptomycin. CLL cells were cultured as total PBMCs or in coculture with stroma in a 5:1 ratio. Cells were stimulated as indicated: either directly with a B cell stimulation cocktail consisting of 25 ng/mL interleukin (IL)-2, IL-15, and IL-21 including 1 µg/mL CpG and/or indirectly via a T cell stimulation of anti-CD3 (clone 1XE) and anti-CD28 (clone 15E8) antibodies.

### Primary LN fibroblasts

Primary LN fibroblasts were a kind gift of T. de Jong and L. van Baarsen (Department of Experimental Immunology, Amsterdam UMC). Inguinal LN needle biopsies were obtained from rheumatoid arthritis patients, healthy controls, and donors with autoantibodies at-risk for developing rheumatoid arthritis. Biopsies were enzymatically digested with dispase, collagenase, and DNAse. Afterwards, fibroblasts were grown out over the course of several weeks of in vitro culture.

### Flow cytometry

For proliferation assays, PBMCs were labeled with either CFSE or CellTrace Violet. After culture, spheroids were resuspended and disintegrated to ensure proper antibody staining. Cells were incubated with monoclonal antibodies for surface staining for 30 minutes at 4°C. Cells were stained with the following antibodies: CD4, CD8, CD14, CD19, CD25, CD56, CD95, and a viability dye (Table 1). Samples were measured on a Canto II flow cytometer (BD Biosciences, Franklin Lakes, NJ). Samples were analyzed using FlowJo software.

**Table 1:** Antibodies

Product	Article Number	Supplier
Patient sample characterization		
Anti-human CD8	CYT-8F8	Cytognos (Madrid, Spain)
Anti-human CD56	CYT-56PE	Cytognos (Madrid, Spain)
Anti-human CD5	341109	BD Biosciences (Franklin Lakes, NJ)
Anti-human CD19	IM3628	Beckman Coulter (Indianapolis, IN)
Anti-human TCRγδ	655410	BD Biosciences (Franklin Lakes, NJ)
Anti-human CD3	345767	BD Biosciences (Franklin Lakes, NJ)
Anti-human CD4	300524	BioLegend (San Diego, CA)
Anti-human CD20	302320	BioLegend (San Diego, CA)
Flow cytometry		
Anti-human CD4	46-0047-42	eBioscience (Santa Clara, CA)
Anti-human CD8	344732	BioLegend (San Diego, CA)
Anti-human CD14	340436	BD Biosciences (Franklin Lakes, NJ)
Anti-human CD19	555412	BD Biosciences (Franklin Lakes, NJ)
Anti-human CD25	R0811	Dako (Santa Clara, CA)
Anti-human CD56	CYT-56PE	Cytognos (Madrid, Spain)
Anti-human CD95	305621	BioLegend (San Diego, CA)
Confocal microscopy		
Alexa Fluor 488 anti-human CD4	300519	BioLegend (San Diego, CA)
Alexa Fluor 594 anti-human CD8a	301056	BioLegend (San Diego, CA)
Alexa Fluor 647 anti-human CD14	325612	BioLegend (San Diego, CA)
Alexa Fluor 700 anti-human CD19	302226	BioLegend (San Diego, CA)

CA = California; IN = Indiana; NJ = New Jersey.

### Confocal microscopy

During culture, spheroids were imaged using standard light field microscopy. After culture, the supernatant was removed, and spheroids were embedded in 1% agarose to preserve spheroid structure. Agarose-embedded spheroids were then fixated in 4% paraformaldehyde for 10 minutes at room temperature, permeabilized in 0.01% Triton-X for 10 minutes at room temperature, blocked in 0.5% BSA for 1 hour at room temperature, and washed with PBS in between steps. Immunofluorescent staining of spheroids was carried out via incubation with primary antibodies overnight at 4°C and incubation with secondary antibodies for 1 hour at room temperature while washing with PBS in between steps. Cells were stained with the following antibodies: CD4-AlexaFluor488, CD8-AlexaFluor549, CD14-AlexaFluor647, and CD19-AlexaFluor700 (Table 1). Afterwards, cells were stained in DAPI solution for 10 minutes at room temperature. Finally, agarose-embedded spheroids were transferred to an 18-well glass bottom microscopic slide and analyzed using an SP8-X SMD confocal microscope (Leica Microsystems, Wetzlar, Germany).

### Kinetic growth assays

The 3D cultures were stimulated and treated as indicated. Acalabrutinib and ibrutinib were purchased from Selleck Chemicals (Houston, Texas). Culture plates were placed in an IncuCyte live-cell imager (Essen Biosciences, Royston, United Kingdom) in an incubator at 37°C and 5% CO_2_. Scans were taken every 12 hours using the single spheroid assay for live-cell analysis application using ×4 magnification. Spheroid area was quantified using IncuCyte software as a proxy for spheroid growth.

### Venetoclax sensitivity assay

CLL cells were cultured as indicated for 24 hours. After culture, cells were resuspended and incubated with and without venetoclax for an additional 24 hours. Venetoclax was purchased from LKT Laboratories (St Paul, Minnesota). Culture plates were placed in an IncuCyte live-cell imager (Essen Biosciences, Royston, United Kingdom) in an incubator at 37°C and 5% CO_2_. Scans were taken every 1 hour using the single spheroid assay for live-cell analysis application using a ×4 magnification. CLL cell viability was measured by flow cytometry using DioC6 and TO-PRO-3 viability dyes. Samples were measured on a FACS Calibur flow cytometer (BD Biosciences, Franklin Lakes, NJ). Samples were analyzed using FlowJo software. Specific apoptosis is defined as (% cell death in treated cells) – (% cell death in medium control)/(% viable cells medium control) × 100%.

### T cell cytotoxicity assay

Primary CLL cells were labeled with CellTrace Violet according to the manufacturer’s instructions and cocultured with healthy donor T cells in a 4:1 effector to target ratio for 24 hours. PBMCs were incubated in the presence of either 1 ng/mL blinatumomab, 10 µM venetoclax, or medium. The viability of the target cells was assessed by flow cytometry using TO-PRO-3 and MitoTracker Orange viability dyes. Samples were measured on an LSR Fortessa flow cytometer (BD Biosciences, Franklin Lakes, NJ). Specific lysis is defined as (% target cell death in treated sample) – (% target cell death in medium control)/(100% − % target cell death in medium control) × 100%.

### Statistics

Statistical analysis was performed using GraphPad Prism 9 software. A paired *t* test was used to analyze paired observations between 2 groups. Two-way ANOVA with multiple comparisons and Tukey correction for multiple comparisons was used to analyze differences between 3 or more groups. In the case of drug titrations, Geisser-Greenhouse correction was added in combination with Tukey correction for multiple comparisons to analyze differences between groups using 2-way ANOVA; *P < 0.05; **P < 0.01; ***P < 0.001; ****P < 0.0001.

## RESULTS

### Design of a PBMC-based LN model

**Figure 1.**
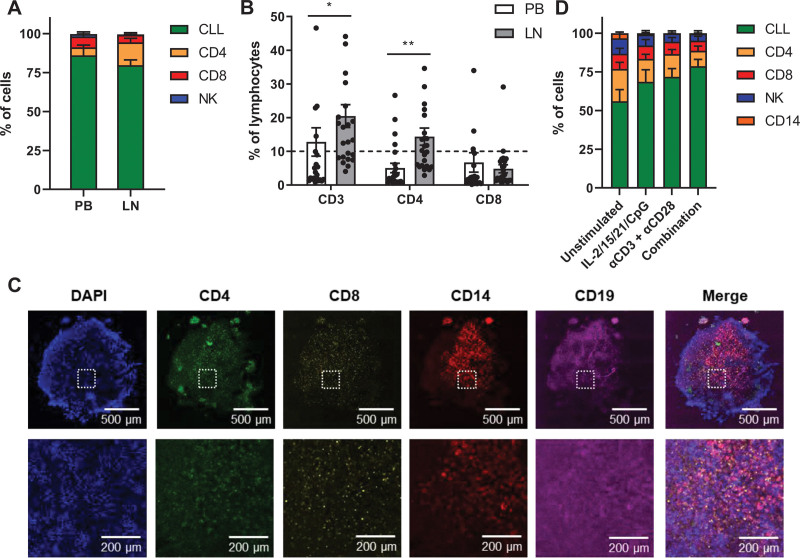
**Design of a PBMC-based LN model.** (A and B) Lymphocytes were isolated from paired PB and LN samples and characterized in treatment-naïve CLL patients (n = 24). (A) Cells were gated based on the following surface markers: CLL (CD5+/CD19+), CD4^+^ T cells (CD3+/CD4), CD8^+^ T cells (CD3+/CD4+), and NK cells (CD56+). Healthy B cells (CD5−/CD19+) were excluded from the analysis due to very small populations. Error bars represent the mean ± SEM. (B) Detailed analysis of the T cell subsets. The dashed line represents the >10% CD4^+^ T cell cutoff we applied in selection of PB biobank samples. Error bars represent the mean ± SEM (n = 24), *P < 0.05, **P < 0.01 (2-way repeated measures ANOVA). (C) CLL sample after spheroid formation. The sample was stained for DAPI (blue), CD4 (green), CD8 (yellow), CD14 (red), and CD19 (magenta) and analyzed by confocal microscopy. (D) CLL samples were stimulated as indicated and cultured in ULA plates for 5 d. Cells were gated based on the following surface markers: CLL (CD5+/CD19+), CD4^+^ T cells (CD3+/CD4+), CD8^+^ T cells (CD3+/CD4+), and NK cells (CD56+). Healthy B cells (CD5−/CD19+) were excluded from the analysis due to very small populations. Error bars represent the mean ± SEM (n = 8). CLL = chronic lymphocytic leukemia; IL = interleukin; LN = lymph node; NK = natural killer; PB = peripheral blood; PBMCs = peripheral blood mononuclear cells; ULA = ultra-low attachment.

As CD4^+^CD40L^+^ T cells have been shown to cluster near Ki-67^+^ CLL cells within LN tissue biopsies,^8^ we hypothesized that improved cell-cell interactions in a 3D conformation aid CLL cells to proliferate and evade cell death.^3,9^ First, we aimed to incorporate T cells as a source of physiological CD40L by recapitulating the composition of CLL and T cells in the CLL LN. To this end, we confirmed that LN samples contained significantly more T cells compared with PB samples, which was specifically due to the increased number of CD4^+^ T cells in the LN (Figure 1A and 1B).^10^ For this reason, we selected PB samples from our CLL biobank containing >10% CD4^+^ T cells to mimic CD4^+^CD40L^+^ T cell help in the LN using primary PBMCs.

After spheroid formation and self-organization of cells, we observed several follicles of CD4^+^ T cells in both the spheroid center and periphery, a population of CD8^+^ T cells that was evenly distributed throughout the spheroid and a network of CD14 myeloid cells around the spheroid center (Figure 1C). To further characterize the model, we investigated the cell composition of the CLL spheroids after 5 days of in vitro culture upon different modes of stimulation. As multiple cell types in the LN microenvironment may affect drug resistance and proliferation of CLL cells via cytokine secretion,^1^ we optimized a cocktail to directly stimulate the CLL cells consisting of IL-2, IL-15, IL-21, and the TLR9 agonist CpG (Suppl. Figure S1). Alternatively, we applied T cell stimulation using αCD3 and αCD28 antibodies, whereas unstimulated spheroids contained ≈50% CLL cells and 20% CD4^+^ T cells, upon either mode of stimulation the CLL fraction expanded to ≈70% (Figure 1D). Consistently, the combination of both stimulations resulted in a further expansion of CLL cells. To keep the system as simple as possible and as complex as necessary, for most readouts presented here, we only applied IL-2/15/21/CpG stimulation unless indicated otherwise.

### 3D cultures promote CLL proliferation in a T cell-dependent manner

**Figure 2.**
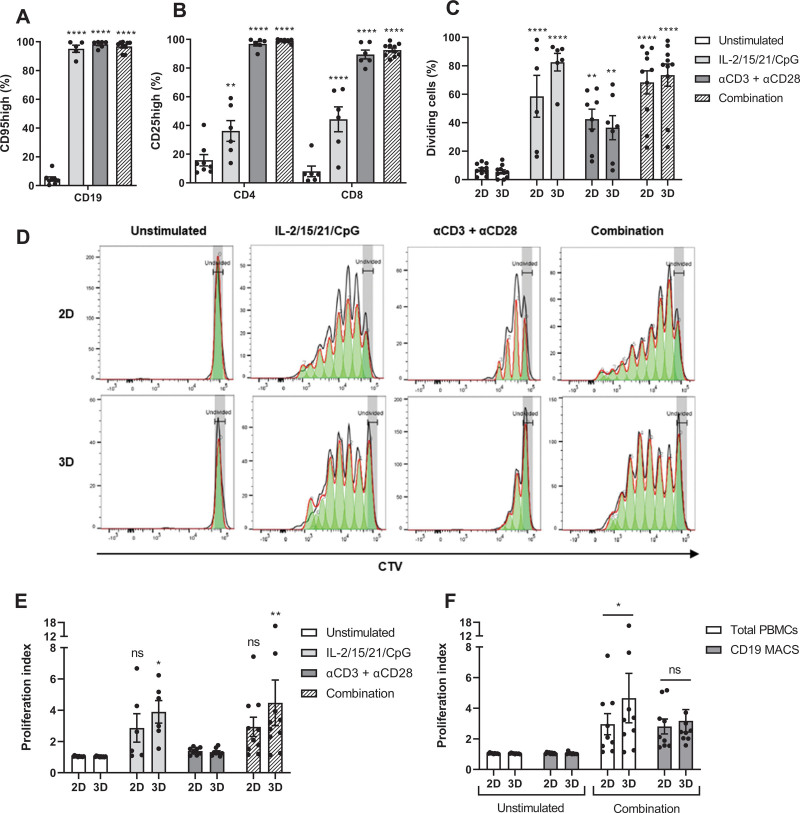
**3D cultures promote CLL proliferation in a T cell-dependent manner.** (A and B) CLL samples were stimulated as indicated and cultured in ULA plates for 5 d. Afterwards, cells were measured using flow cytometry with the CD95 activation marker for CLL cells (A) and CD25 as the activation marker for CD4^+^ or CD8^+^ T cells (B). Error bars represent the mean ± SEM (n = 6 for IL-2/15/21/CpG; n = 6 for αCD3 + αCD28; n = 10 for combination), **P < 0.01, ****P < 0.0001 ([A]: paired *t* test; [B]: 2-way ANOVA). Each group was compared with the unstimulated control group. (C–E) CTV-labeled CLL samples were stimulated as indicated and cultured in standard round-bottom 96-well plates (2D) or ULA plates (3D) for 8 d and measured using flow cytometry. Results of the CD5^+^CD19^+^ CLL population are shown (C and D). Afterwards, the proliferation index of the CD5^+^CD19^+^ CLL population was quantified (E). Bars represent the mean ± SEM (n = 6 for IL-2/15/21/CpG; n = 8 for αCD3 + αCD28; n = 10 for combination), *P < 0.05; **P < 0.01; ****P < 0.0001 (2-way ANOVA). Each group was compared with the unstimulated control group. (F) Stimulation of IL-2/15/21/CpG and αCD3/αCD28 antibodies was performed on the total pool of PBMCs and in parallel on the CD19^+^ CLL fraction without the presence of T cells. The proliferation index of the CD5^+^CD19^+^ CLL population was quantified. Error bars represent the mean ± SEM (n = 9), *P < 0.05 (2-way repeated measures ANOVA). CLL = chronic lymphocytic leukemia; CTV = CellTrace^™^ Violet; MACS = magnetic-activated cell sorting; PBMCs = peripheral blood mononuclear cells; ULA = ultra-low attachment; 2D = two-dimensional; 3D = three-dimensional.

As a marker for responsiveness of CLL cells to soluble stimuli and cell-cell interactions within 3D cultures, we measured the expression of CD95. Stimulation with IL-2/15/21/CpG induced maximal CLL activation (Figure 2A). T cell stimulation via αCD3/αCD28 antibodies also resulted in maximal activation of CLL cells, thereby demonstrating the interactions of CLL and T cells in the model. Yet, T cells required stimulation with αCD3/αCD28 antibodies for 100% activation (Figure 2B). Direct stimulation of CLL cells with IL-2/15/21/CpG resulted in an induction of CLL proliferation with 60% dividing CLL cells in 2D cultures and 80% in 3D cultures (Figure 2C). Stimulation of T cells via αCD3/αCD28 antibodies also triggered CLL proliferation with 40% dividing CLL cells, but dividing cells only proliferated once or twice (Figure 2D). Combination of these 2 modes of stimulation resulted in ≈70% dividing CLL cells in both 2D and 3D cultures, yet 3D cultures showed a significantly increased proliferation index upon stimulation in contrast to 2D cultures (IL-2/15/21/CpG: *P* = 0.0297; combination: *P* = 0.0012) (Figure 2E). This is a result of most cells proliferating once or twice in 2D cultures, whereas 3D cultures showed a similar number of cells in each generation (Figure 2D). Moreover, direct comparison of 2D and 3D cultures indicates a significantly increased proliferation index in 3D cultures (Figure 2F).

To investigate the incorporation of additional cell types into the model, we performed experiments using LN-derived fibroblasts obtained from non-CLL donors. In combination with IL-2/15/21/CpG stimulation, LN fibroblasts increased proliferation in every CLL sample (Suppl. Figure S3A). These results indicate that introduction of additional cell types may further influence the culture of CLL cells. Due to the scarcity of primary LN material, we further employed the model here without the addition of LN fibroblasts. Finally, we investigated the contribution of T cells to the proliferation of CLL cells. Analysis of T cell proliferation shows that only direct T cell stimulation via αCD3/αCD28 antibodies resulted in cell division of all T cells in addition to a significantly increased proliferation index (Suppl. Figure S2). The significantly increased proliferation index observed in 3D cultures was abrogated in the absence of T cells (Figure 2F). These data suggest that 3D cultures promote CLL proliferation via T cells.

### 3D cultures enable the long-term proliferation of CLL cells with the emergence of follicle-like structures

**Figure 3.**
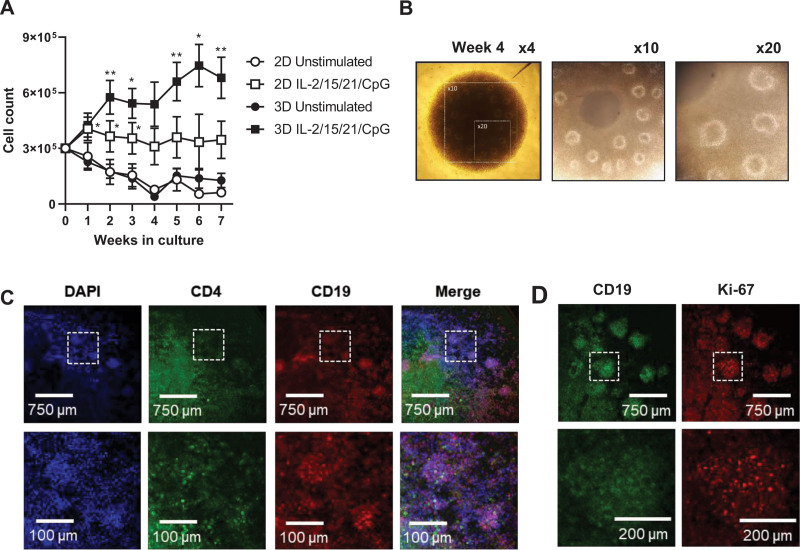
**3D cultures enable the long-term proliferation of CLL cells.** (A–D) CLL samples were stimulated as indicated and cultured in standard round-bottom 96-well plates (2D) or ULA plates (3D) for 7 wks. (A) Total cell counts were measured every week by flow cytometry. Error bars represent the mean ± SEM (n = 6), *P < 0.05, **P < 0.01 (paired *t* test). Each group was compared with the unstimulated control group. (B) Spheroid morphology observed by light field microscopy. The panels show× 4, ×10, and ×20 magnifications of the spheroid at week 4. (C) Spheroids were cultured for 4 wks and stained for DAPI (blue), CD4 (green) and CD19 (red). Spheroids were imaged by confocal microscopy, including magnifications in the bottom panel. (D) Spheroids were cultured for 4 wks and stained for CD19 (green) and Ki-67 (red). Spheroids were imaged by confocal microscopy, including magnifications in the bottom panel. CLL = chronic lymphocytic leukemia; SEM = standard error of mean; ULA = ultra-low attachment; 2D = two-dimensional.

Next, we investigated the longevity of CLL cultures after stimulation with IL-2/15/21/CpG. For this purpose, we did not apply αCD3/αCD28 antibody stimulation, as then T cells rapidly proliferate and dominate the culture. After 1 week of culture, stimulated 3D cultures expanded in a linear fashion with a 2-fold expansion at 2 weeks of culture, whereas cell numbers in 2D cultures started to collapse and eventually reached a plateau (Figure 3A). In the 3D cultures, a second expansion phase occurred at around 4 weeks. Interestingly, spheroids started to develop follicle-like structures around 3–4 weeks of culture (Figure 3B). We observed that CD4^+^ T cells localized both at the spheroid core and in these follicle-like structures (Figure 3C). Furthermore, follicle-like structures showed an enrichment of Ki-67^+^ CLL cells, suggesting that these are also sites of proliferation (Figure 3D). These data show that 3D cultures enable the expansion and long-term proliferation of CLL cells, with emergence of self-organizing, follicle-like structures.

### Bruton's tyrosine kinase (Btk) targeting inhibits spheroid growth and impacts spheroid architecture

**Figure 4.**
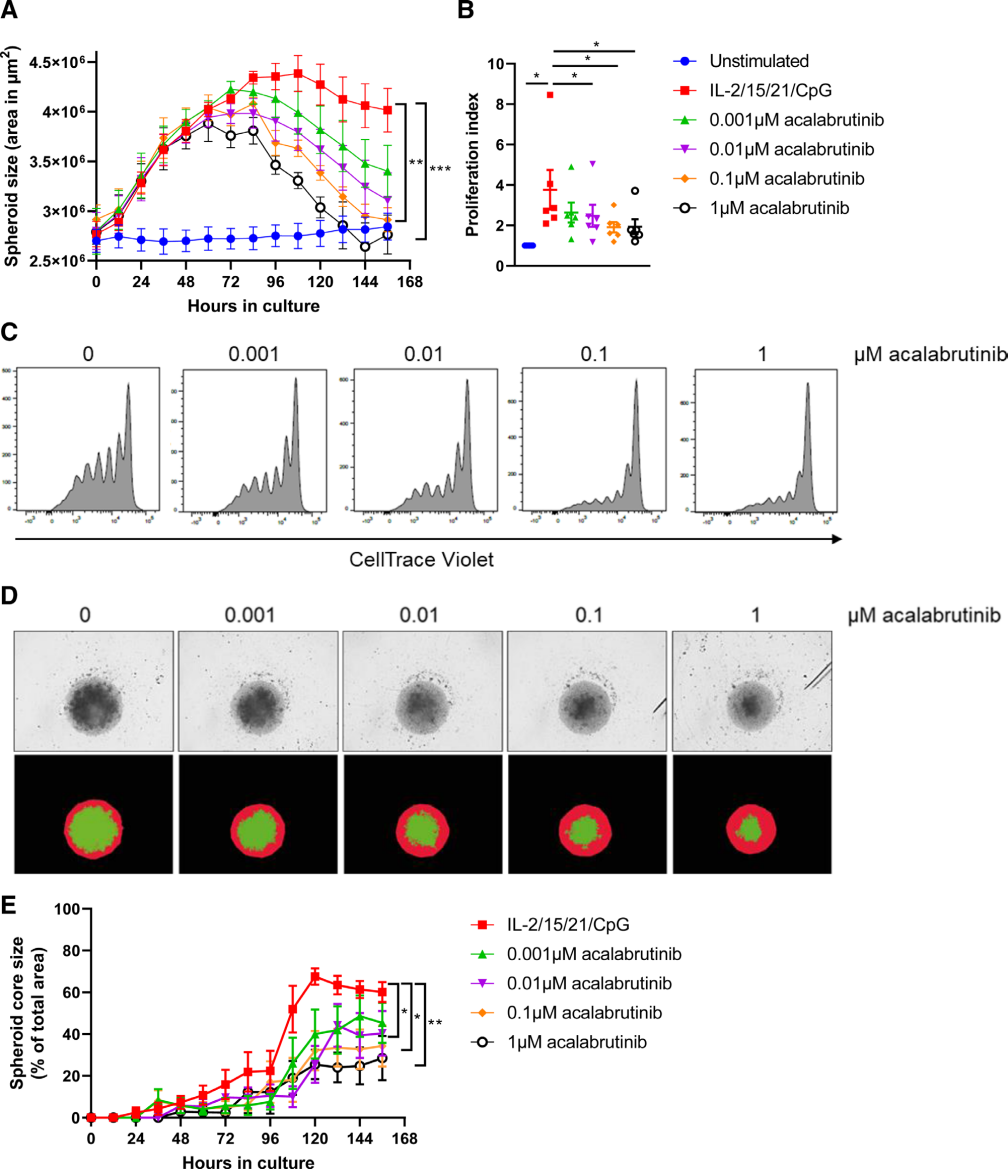
**Btk targeting inhibits spheroid growth and impacts spheroid architecture.** (A–C) CTV-labeled CLL samples were stimulated with IL-2/15/21/CpG and cultured in ULA plates (3D) for 6 d in the presence of 0.001–1 µM acalabrutinib. Culture plates were placed in an IncuCyte live-cell imager, which imaged and quantified the spheroid area every 12 h (A). Error bars represent the mean ± SEM (n = 6), **P < 0.01, ***P < 0.001 (2-way ANOVA). Afterwards, cells were measured using flow cytometry (C), and the proliferation index of the CD5^+^CD19^+^ CLL population was quantified (B). Error bars represent the mean ± SEM (n = 6), *P < 0.05 (paired *t* test). (D and E) Based on the live-cell imaging data, spheroid morphology was analyzed by quantifying the total spheroid area (red mask) and quantifying the spheroid core area (green mask) (D). The spheroid core area was then plotted over time as a percentage of the total spheroid area, where 100% equals to an identical area of red and green masks (E). Error bars represent the mean ± SEM (n = 6), *P < 0.05, **P < 0.01 (2-way repeated measures ANOVA). CLL = chronic lymphocytic leukemia; CTV = CellTrace^™^ Violet; SEM = standard error of mean; ULA = ultra-low attachment.

A 3D model enables novel assays and readouts such as kinetic growth assays using live-cell imaging. We performed a proliferation experiment in the presence of the Btk inhibitor acalabrutinib and imaged spheroids in real-time from which the spheroid area was quantified as a proxy for spheroid growth. Independent of the presence of acalabrutinib, spheroids stimulated with IL-2/15/21/CpG showed an initial growth to 4 × 10^6^ µm^2^ over the course of 3 days (Figure 4A). Although untreated spheroids reached a plateau in spheroid growth, acalabrutinib treatment decreased spheroid size in a dose-dependent manner, eventually reaching the size of unstimulated spheroids upon 0.1–1 µM acalabrutinib. No significant differences were observed in terms of spheroid growth and response to acalabrutinib treatment between immunoglobulin heavy chain variable region (IGHV)-mutated and nonmutated CLL patients (Suppl. Figure S4A). To test the accuracy of this assay as a readout for cell proliferation, we subjected the imaged spheroids to standard flow cytometry as an end point readout. Based on the CellTrace Violet signal, the proliferation index was quantified in each spheroid, confirming significantly decreased proliferation (1 µM acalabrutinib: *P* = 0.0348) in a dose-dependent manner (Figure 4B and 4C). Upon acalabrutinib treatment, we observed no significant effects on CLL cell viability or activation (Suppl. Figure S4B and S4C). Although both IncuCyte and flow cytometry readouts were consistent, the IncuCyte assay provided not only real-time data, but also seemed more sensitive compared with flow cytometry. Notably, acalabrutinib treatment affected not only spheroid size, but also impacted spheroid architecture (Figure 4D). As a proxy for cell-cell adhesion, we plotted the area of the spheroid core as a percentage of total spheroid area, where a lower percentage translates to more detached cells. Stimulation of spheroids with IL-2/15/21/CpG resulted in the formation of a dense spheroid core structure consisting of adherent cells, expanding to ≈70% of the total spheroid area as a result of proliferation. Acalabrutinib treatment was associated with a lower core expansion (30%–40%) and increased areas of low-density regions located at the outer spheroid edges, consisting of detaching cells that can easily be dissociated (Figure 4E).

### CLL spheroids enable the study of drug resistance and T cell cytotoxicity

**Figure 5.**
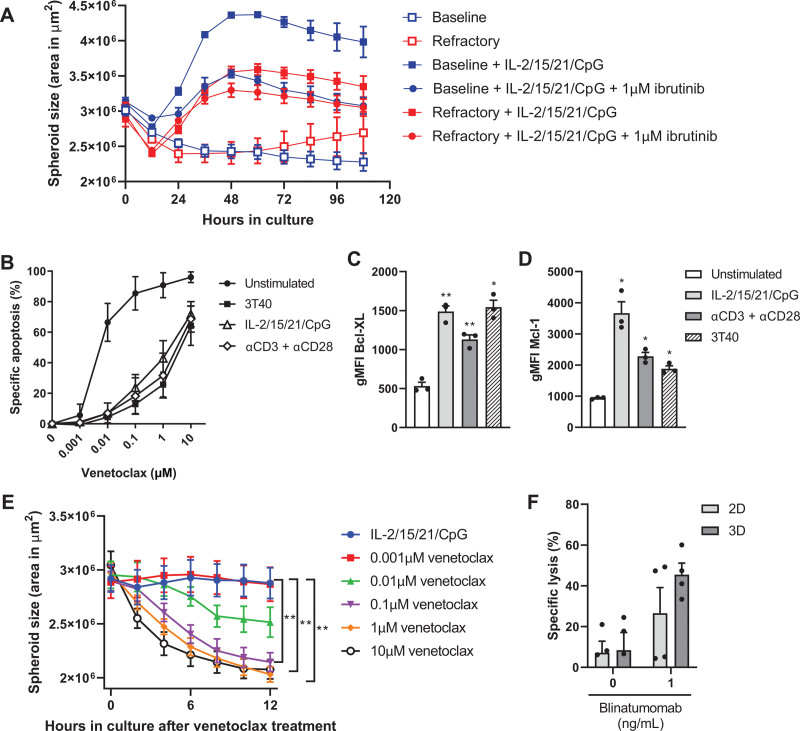
**CLL spheroids enable the study of drug resistance and T cell cytotoxicity.** (A) Paired baseline and refractory samples of an ibrutinib-treated CLL patient were stimulated with IL-2/15/21/CpG and cultured in ULA plates (3D) for 4 d in the presence of 1 µM ibrutinib. Culture plates were placed in an IncuCyte live-cell imager, which imaged and quantified the spheroid area every 12 h. The data represents 9 spheroids from 2 patients cultured in each condition. Error bars represent the mean ± SEM. (B–E) CLL samples were stimulated as indicated and/or cocultured with 3T40 fibroblast in ULA plates (3D) for 1 wk. Afterwards, cells were incubated with a titration of 0.001–10 µM venetoclax for an additional 24 h, during which the culture plates were placed in an IncuCyte live-cell imager, which imaged and quantified spheroid area every hour. The data represents 7 spheroids from 4 patients cultured in each condition (E). Viability was measured by flow cytometry using DiOC6 and TO-PRO-3 viability dyes (B), including expression of Bcl-XL (C) and Mcl-1 (D). Error bars represent the mean ± SEM (n = 3), *P < 0.05, **P < 0.01 (paired *t* test). Each group was compared with the unstimulated control group. (F) CTV-labeled CLL cells were cocultured with healthy donor T cells in a 4:1 effector to target ratio in the presence of 1 ng/mL blinatumomab for 24 h. Viability was measured by flow cytometry using MitoTracker Orange and TO-PRO-3 viability dyes. Error bars represent the mean ± SEM (n = 4). CLL = chronic lymphocytic leukemia; CTV = CellTrace^™^ Violet; IL = interleukin; SEM = standard error of mean; ULA = ultra-low attachment; 2D = two-dimensional; 3D = 3-dimensional.

Furthermore, this 3D CLL model can be used to investigate both in vitro and in vivo drug resistance. As an example, we performed a proliferation experiment using paired baseline and refractory samples of 2 CLL patients who relapsed on ibrutinib monotherapy. Baseline samples showed strong inhibition of spheroid growth upon in vitro ibrutinib treatment, whereas spheroid growth of refractory samples was barely affected by in vitro ibrutinib treatment (Figure 5A). Notably, in 1 of the 2 patients, refractory cells displayed spheroid growth without any in vitro stimulation. The 3D model described here can also be used for more traditional cytotoxic drug screening using either a flow cytometry or live-cell imaging readout. Direct stimulation of CLL cells with IL-2/15/21/CpG resulted in venetoclax sensitivity similar to 3D coculture with 3T40 fibroblasts, reaching a maximum of 70% specific apoptosis upon 10 µM venetoclax (Figure 5B). Stimulation of T cells using αCD3/αCD28 resulted in comparable venetoclax sensitivity, indicating that T cells are able to confer protection to CLL cells in this system, which was associated with the upregulation of the anti-apoptotic Bcl-2 family members Bcl-XL and Mcl-1 (Figure 5C and 5D). Notably, coculture with 3T40 fibroblasts resulted in a major shift in venetoclax sensitivity, whereas coculture with primary LN fibroblasts did not (Suppl. Figure S3B). Aside from proliferation studies, live-cell imaging can also be applied for large-scale drug screening, thereby providing real-time data in addition to endpoint analysis. Treatment with 0.1–10 µM venetoclax significantly decreased spheroid size in a dose-dependent manner (Figure 5E). Furthermore, as proof-of-principle, we performed a T cell cytotoxicity assay via coculture of healthy donor T cells with CLL cells in the presence of the CD3xCD19 bispecific antibody blinatumomab. Coculture in a 4:1 effector to target ratio resulted in specific lysis of CLL cells of 26% in 2D cultures versus 45% in 3D cultures. Notably, 2 healthy donor samples did not respond in standard 2D cultures, whereas they did demonstrate cytotoxicity within the CLL spheroids, thus potentially promoting T cell killing capacity in a 3D conformation (Figure 5F). These data show that conventional T cell cytotoxicity assays are compatible with 3D cultures, thereby opening up new immunotherapies that can be tested in this 3D model, such as antibodies, bispecifics, and CAR T cells.

## DISCUSSION

We developed a 3D culture model using PBMCs from CLL patients, with incorporation of key aspects of CLL LN biology. Based on the 3T40-CLL coculture model,^11^ we used T cells as a source of physiological CD40L by mimicking the CLL:T cell composition of the CLL LN in vitro. By using ULA plates, we created a model that only requires common laboratory equipment, which can thus easily be adopted by other research groups. Using only soluble factors for CLL cell stimulation, the IL-2/15/21/CpG cocktail we presented here can easily be adapted for more specialized applications, such as the αCD3/αCD28 T cell stimulation we also presented. Furthermore, the low required number of cells for seeding and the compatibility with 1 medium exchange per week in the case of long-term cultures makes this model practical to use. Finally, the use of a 96-well plate format enables high-throughput drug screening and still allows accurate comparison with standard 2D culture models.

Our data indicate that enhanced CLL proliferation in 3D cultures is dependent on the presence of T cells. This may be a result of improved interactions of CLL cells with surrounding T cells in a 3D conformation. Noteworthy, although the proliferation index increased in 3D cultures, the maximum number of generations remained the same compared with 2D cultures, suggesting that CLL cells reached a plateau after a certain number of cell divisions. We confirmed that coculture of CLL cells with activated T cells may trigger CLL proliferation,^3^ although proliferating CLL cells showed a limited number of cell divisions. A potential explanation is that CD40 stimulation primes CLL cells for proliferation but that additional signals are needed to sustain a proliferative phenotype and drive CLL cells through several cell cycles, such as costimulation with cytokines.^3^ Although T cell stimulation does induce the secretion of relevant T cell cytokines, the production and resulting cytokine levels in vitro may not be sufficient to sustain CLL proliferation.

Regarding the physiological relevance of the included cytokines and TLR stimuli, we based our stimulation cocktail on the presence of the available cell types in the LN and the cytokines that they secrete and their effects on CLL proliferation in previous in vitro studies, in combination with signaling pathways found to be active in LN-derived CLL cells. IL-21 is produced by CD4^+^ T cells and follicular T cells, and primes CLL cells for proliferation but also renders them more sensitive to apoptosis.^3^ IL-15 is produced by monocytes and may enhance CLL proliferation and survival.^12^ IL-2 shares these properties and is primarily produced by CD4^+^ and CD8^+^ T cells.^13,14^ Importantly, CD40 activation upregulates the IL-21, IL-15, and IL-2 receptors,^15,16^ which may further prime CLL cells for proliferation and survival. Finally, we applied CpG as a ligand to activate TLR signaling in CLL cells, as is shown by gene expression studies in LN-derived CLL cells,^17,18^ which may further promote the upregulation of cytokine receptors.^19^ Although the exact in vivo TLR9 ligand remains unknown, a recent study indicated a role of cell-free mitochondrial DNA in plasma from CLL patients, which can trigger TLR9 signaling in a similar fashion to CpG.^20^

Acalabrutinib treatment inhibited spheroid growth and was associated with morphological changes, potentially due to inhibition of Btk-mediated cell adhesion leading to the dissociation of adherent cells. An interesting aspect is how Btk inhibition could affect spheroid growth without the addition of direct B cell receptor (BCR) stimulation. First, it has been shown that Btk is involved in both TLR-mediated and CD40-mediated CLL proliferation.^21–23^ Second, Btk is involved in cell adhesion,^24^ and inhibition of Btk may thus affect spheroid growth by disrupting cell-cell interactions within the spheroid. Furthermore, our data demonstrate Bcl-XL and Mcl-1 upregulation by CLL cells after either IL-2/15/21/CpG or T cell stimulation, similar to what is observed in CLL LN samples,^11,25^ thereby adding physiological relevance. This could enable ibrutinib-venetoclax synergy studies in an ex vivo model. Finally, we observed enhanced CLL proliferation in cocultures with LN-derived stromal cells. The use of CLL-derived or even autologous LN stroma may further support CLL proliferation in vitro. Although such material is rare, our data suggest that this 3D model provides a foundation that allows the introduction of additional cell types to the culture and can thus be used for the generation of more complex microenvironments. Moreover, the addition of other cell types such as follicular dendritic cells or follicular T cells^26,27^ could further influence structure, proliferation, and drug resistance. A distinctive characteristic of CLL LN tissues is the presence of pseudofollicles.^28,29^ These are proliferation centers that have not been described in other lymphoproliferative conditions and are enriched in CD40L^+^ T cells and Ki-67^+^ CLL cells compared with surrounding tissue.^30^ The emergence of follicle-like structures after prolonged culture in the model could indicate a similar phenotype. Although the presence of follicles can be beneficial by allowing efficient cell-cell communication, this could also result in malnutrition and hypoxia, leading to cell death.^31^

An obvious limitation of the model presented here is the requirement to characterize CLL samples and select them based on the CLL:T cell composition. Alternatively, PBMCs can be sorted to acquire the desired CLL:T cell composition. Here, we applied a >10% CD4^+^ T cell cutoff based on the LN biopsy analysis, but it remains unknown where the actual threshold lies in which 3D cultures do not show increased CLL proliferation compared with 2D cultures. Nevertheless, spheroid growth assays can still be carried out independent of CLL:T cell composition, as shown for the ibrutinib-relapse sample that we demonstrated here. A potential oversight of the model is the lack of in vitro BCR stimulation using αIgM antibodies, which has been shown to enhance in vitro CLL proliferation in combination with costimulatory signals, including a combination of CD40 and cytokine signaling.^32^ Although BCR signaling has been acknowledged as a key mechanism for CLL progression in vivo,^33^ we avoided the use of in vitro BCR stimulation to exclude differences between IGHV-mutated and IGHV-nonmutated CLL samples. It should be noted, however, that CLL cells are capable of autonomous BCR signaling, a feature that has been attributed to the CLL BCR.^34,35^

In summary, we present a novel culture system that underlines the role of T cells in CLL proliferation and is capable of sustained growth of CLL cells. In addition to being a scalable, multipurpose platform, this model may enable the investigation of the outgrowth of genetic (sub)clones^36,37^ and spatial phenotyping of both tumor and nonmalignant cells, thereby uncovering new biomarkers and targets for therapy. The model described here permits investigation of CLL cells in the context of their natural protective niche and what role this support plays in CLL drug resistance, thereby opening many new avenues for clinically useful applications.

## ACKNOWLEDGMENTS

The authors thank Tineke de Jong and Lisa van Baarsen for providing primary lymph node fibroblasts for the experiment shown in Suppl. Figure S3.

## AUTHOR CONTRIBUTIONS

MVH designed the study, performed research, analyzed data, and wrote the article. BFvD and EP performed research. DdR processed and characterized patient samples and analyzed data. DL analyzed data and critically reviewed the article. RL and PDM acquired financial support and critically reviewed the article. AP provided patient samples and wrote the article. EE designed the study, acquired financial support, and wrote the article.

## DISCLOSURES

RL is an employee and equity holder of Bristol Myers Squibb. All the other authors have no conflicts of interest to disclose.

## SOURCES OF FUNDING

This project CLL Lymph node Organoids for Screens and Intelligent Testing (CLOSIT) is in collaboration with Acerta Pharma and Bristol Myers Squibb. The collaboration project is financed by the Ministry of Economic Affairs by means of the PPP Allowance made available by the Top Sector Life Sciences and Health to stimulate public-private partnerships.

## Supplementary Material


