# Supplementary material for: In Vitro 3D Spheroid Culture System Displays Sustained T Cell-dependent CLL Proliferation and Survival
Source: Hemasphere. 2023 Aug 23;7(9):e938. doi: 10.1097/HS9.0000000000000938 (PMC10448932; doi:10.1097/HS9.0000000000000938)
Supplement: Supplementary file 1 [file hs9-7-e938-s001.pdf]

sample ID	experiment	sample date	age	gender	IgHV mutation status	treatment	article figure
H1580001T1	Biobank (PB)	30-12-2020	74	M	unknown	none	1A-B
H1580001T1	Biobank (LN)	30-12-2020	74	M	unknown	none	1A-B
H1580002T1	Biobank (PB)	4-1-2021	52	M	unknown	none	1A-B
H1580002T1	Biobank (LN)	4-1-2021	52	M	unknown	none	1A-B
H1580004T1	Biobank (PB)	18-1-2021	59	M	unknown	none	1A-B
H1580004T1	Biobank (LN)	18-1-2021	59	M	unknown	none	1A-B
H1580005T1	Biobank (PB)	19-1-2021	72	M	unknown	none	1A-B
H1580005T1	Biobank (LN)	19-1-2021	72	M	unknown	none	1A-B
H1580006T1	Biobank (PB)	19-1-2021	74	F	unknown	none	1A-B
H1580006T1	Biobank (LN)	19-1-2021	74	F	unknown	none	1A-B
H1580009T1	Biobank (PB)	27-1-2021	60	M	unknown	none	1A-B
H1580009T1	Biobank (LN)	27-1-2021	60	M	unknown	none	1A-B
H1580010T1	Biobank (PB)	3-2-2021	64	M	unknown	none	1A-B
H1580010T1	Biobank (LN)	3-2-2021	64	M	unknown	none	1A-B
H1580011T1	Biobank (PB)	8-2-2021	70	M	unknown	none	1A-B
H1580011T1	Biobank (LN)	8-2-2021	70	M	unknown	none	1A-B
H1580016T1	Biobank (PB)	2-3-2021	82	M	unknown	none	1A-B
H1580016T1	Biobank (LN)	2-3-2021	82	M	unknown	none	1A-B
H1580018T1	Biobank (PB)	23-2-2021	71	M	unknown	none	1A-B
H1580018T1	Biobank (LN)	23-2-2021	71	M	unknown	none	1A-B
H1580019T1	Biobank (PB)	24-2-2021	35	M	unknown	none	1A-B
H1580019T1	Biobank (LN)	24-2-2021	35	M	unknown	none	1A-B
H1580021T1	Biobank (PB)	25-2-2021	66	M	unknown	none	1A-B
H1580021T1	Biobank (LN)	25-2-2021	66	M	unknown	none	1A-B
H1580023T1	Biobank (PB)	3-3-2021	65	M	unknown	none	1A-B
H1580023T1	Biobank (LN)	3-3-2021	65	M	unknown	none	1A-B
H1580025T1	Biobank (PB)	2-3-2021	68	M	unknown	none	1A-B
H1580025T1	Biobank (LN)	2-3-2021	68	M	unknown	none	1A-B
H1580028T1	Biobank (PB)	16-3-2021	60	F	unknown	none	1A-B
H1580028T1	Biobank (LN)	16-3-2021	60	F	unknown	none	1A-B
H1580029T1	Biobank (PB)	9-3-2021	67	F	unknown	none	1A-B
H1580029T1	Biobank (LN)	9-3-2021	67	F	unknown	none	1A-B
H1580030T1	Biobank (PB)	15-3-2021	66	F	unknown	none	1A-B
H1580030T1	Biobank (LN)	15-3-2021	66	F	unknown	none	1A-B
H1580031T1	Biobank (PB)	23-3-2021	68	M	unknown	none	1A-B
H1580031T1	Biobank (LN)	23-3-2021	68	M	unknown	none	1A-B
H1580032T1	Biobank (PB)	15-3-2021	86	M	unknown	none	1A-B
H1580032T1	Biobank (LN)	15-3-2021	86	M	unknown	none	1A-B
H1580033T1	Biobank (PB)	16-3-2021	56	F	unknown	none	1A-B
H1580033T1	Biobank (LN)	16-3-2021	56	F	unknown	none	1A-B
H1580034T1	Biobank (PB)	17-3-2021	58	M	unknown	none	1A-B
H1580034T1	Biobank (LN)	17-3-2021	58	M	unknown	none	1A-B
H1580036T1	Biobank (PB)	24-3-2021	75	M	unknown	none	1A-B
H1580036T1	Biobank (LN)	24-3-2021	75	M	unknown	none	1A-B
H1580038T1	Biobank (PB)	30-3-2021	73	M	unknown	none	1A-B
H1580038T1	Biobank (LN)	30-3-2021	73	M	unknown	none	1A-B
H1580039T1	Biobank (PB)	30-3-2021	63	F	unknown	none	1A-B
H1580039T1	Biobank (LN)	30-3-2021	63	F	unknown	none	1A-B
2967	Confocal staining	6-4-2020	69	F	unknown	none	1C
2367	Immunophenotyping	18-9-2018	68	F	unknown	none	1D
2549	Immunophenotyping	23-4-2019	77	M	unknown	none	1D
3067	Immunophenotyping	26-6-2020	60	F	unknown	none	1D
2218	Immunophenotyping	15-5-2018	51	M	unknown	none	1D
2973	Immunophenotyping	14-4-2020	70	M	unknown	none	1D
3065	Immunophenotyping	24-6-2020	62	F	unknown	none	1D
3236	Immunophenotyping	26-10-2020	70	F	unknown	none	1D
2244	Immunophenotyping	5-6-2018	49	M	unknown	none	1D
1093	CLL/T cell activation/proliferation	5-9-2011	53	M	mutated	none	2A-C, 2E-F, S2A-D
1448	CLL/T cell activation/proliferation	6-2-2014	63	M	mutated	none	2A-C, 2E-F, S2A-D
1093	CLL/T cell activation/proliferation	5-9-2011	53	M	mutated	none	2A-C, 2E-F, S2A-D
1201	CLL/T cell activation/proliferation	11-5-2012	61	F	unknown	none	2A-C, 2E-F, S2A-D
1093	CLL/T cell activation/proliferation	5-9-2011	53	M	mutated	none	2A-F, S2A-E
1201	CLL/T cell activation/proliferation	11-5-2012	61	F	unknown	none	2A-C, 2E-F, S2A-D
1201	CLL/T cell activation/proliferation	11-5-2012	61	F	unknown	none	2A-C, 2E-F, S2A-D
1239	CLL/T cell activation/proliferation	24-7-2012	71	F	unmutated	none	2A-C, 2E-F, S2A-D
1201	CLL/T cell activation/proliferation	11-5-2012	61	F	unknown	none	2A-C, 2E-F, S2A-D
1239	CLL/T cell activation/proliferation	24-7-2012	71	F	unmutated	none	2A-C, 2E-F, S2A-D
333	Long-term proliferation cultures	24-5-2002	86	F	unmutated	none	3A-B
1201	Long-term proliferation cultures	11-5-2012	61	F	unknown	none	3A
2089	Long-term proliferation cultures	16-1-2018	65	F	unknown	none	3A, 3D
1448	Long-term proliferation cultures	6-2-2014	63	M	mutated	none	3A
361	Long-term proliferation cultures	29-8-2000	69	M	unmutated	chloorambucil, 2x fludarabine, CHOP+R, radiotherapy	3A
2089	Long-term proliferation cultures	16-1-2018	65	F	unknown	none	3A
885	Long-term proliferation cultures	7-1-2010	92	F	mutated	none	3C
273	Acalabrutinib proliferation assay	29-6-2006	77	M	mutated	none	4A, S4A-C

1065	Acalabrutinib proliferation assay	8-6-2011	73	M	mutated	none	4A-B, S4A-C
1065	Acalabrutinib proliferation assay	8-6-2011	73	M	mutated	none	4A-C, S4C
2791	Acalabrutinib proliferation assay	11-12-2019	55	M	unknown	none	4B, S4B-C
3194	Acalabrutinib proliferation assay	21-9-2020	62	M	mutated	FCR	4B, S4B-C
222	Acalabrutinib proliferation assay	28-2-2006	54	M	polyclonal	none	4B
1401	Acalabrutinib proliferation assay	24-9-2013	78	M	unmutated	none	4B
273	Cohesion analysis	29-6-2006	77	M	mutated	none	4E
1065	Cohesion analysis	8-6-2011	73	M	mutated	none	4E
2791	Cohesion analysis	11-12-2019	55	M	unknown	none	4D-E
1546	Refractory ibrutinib sample	24-9-2014	50	M	unmutated	FCR	5A
3104	Refractory ibrutinib sample	21-7-2020	56	M	unmutated	FCR, ibrutinib	5A
EE5.1	Refractory ibrutinib sample	6-9-2012	66	?	unknown	none	5A
EE5.3	Refractory ibrutinib sample	30-7-2015	69	?	unknown	ibrutinib	5A
197	Venetoclax sensitivity	15-12-2005	60	M	unmutated	2x chloorambucil, radiotherapy, fludarabine	5B, S3B
1557	Venetoclax sensitivity	17-10-2014	64	F	mutated	none	5B, S3B
1873	Venetoclax sensitivity	16-1-2017	54	F	mutated	none	5B, S3B
64	Intracellular Bcl-2 FACS	16-12-2004	66	F	mutated	none	5C-D
1085	Intracellular Bcl-2 FACS	9-8-2011	70	M	mutated	none	5C-D
2029	Intracellular Bcl-2 FACS	12-9-2017	77	F	mutated	none	5C-D
702	3D cytotoxicity	16-10-2008	64	F	mutated	none	5E
721	3D cytotoxicity	18-12-2008	85	M	mutated	none	5E
708	3D cytotoxicity	13-11-2008	68	F	mutated	none	5E
839	3D cytotoxicity	9-9-2009	71	F	mutated	none	5E
123	T cell cytotoxicity	25-7-2003	61	M	mutated	none	5F
1659	T cell cytotoxicity	25-6-2015	57	M	mutated	none	5F
HD 93	T cell cytotoxicity	1-12-2021	63	M	-	-	5F
HD 96	T cell cytotoxicity	1-12-2021	62	M	-	-	5F
1753	2D-3D proliferation optimization	21-3-2016	62	F	unmutated	FCR, chloorambucil	S1A-C
1797	2D-3D proliferation optimization	30-8-2016	74	F	mutated	none	S1A-C
1439	2D-3D proliferation optimization	13-1-2014	57	M	mutated	none	S1A-C
1673	2D-3D proliferation optimization	3-8-2015	40	M	unmutated	none	S1A-C
1808	2D-3D proliferation optimization	12-9-2016	46	M	unmutated	none	S1A-C
1254	2D-3D proliferation optimization	11-9-2012	62	F	mutated	none	S1A-C
1254	2D-3D proliferation optimization	11-9-2012	62	F	mutated	none	S1A-C
1857	2D-3D proliferation optimization	2-1-2017	84	M	unmutated	none	S1A-C
2038	2D-3D proliferation optimization	2-10-2017	68	F	mutated	none	S1A-C
187	LN stroma proliferation	15-11-2005	58	F	mutated	none	S3A
197	LN stroma proliferation	15-12-2005	60	M	unmutated	2x chloorambucil, radiotherapy, fludarabine	S3A
1673	LN stroma proliferation	3-8-2015	40	M	unmutated	none	S3A
H141-009	Acalabrutinib proliferation assay	4-9-2017	52	F	mutated	chloorambucil, ofatumumab	S4A
267	Acalabrutinib proliferation assay	19-6-2006	61	M	unmutated	2x chloorambucil, radiotherapy, fludarabine	S4A
667	Acalabrutinib proliferation assay	20-6-2008	54	M	unmutated	2x chloorambucil + rituximab, chloorambucil, ibrutinib, ofatumumab	S4A
667	Acalabrutinib proliferation assay	20-6-2008	54	M	unmutated	2x chloorambucil + rituximab, chloorambucil, ibrutinib, ofatumumab	S4A
247	Acalabrutinib proliferation assay	2-5-2006	27	F	unmutated	fludarabine, FC + alemtuzumab	S4A
1546	Acalabrutinib proliferation assay	24-9-2014	50	M	unmutated	FCR, ibrutinib, venetoclax	S4A
64	Acalabrutinib proliferation assay	16-12-2004	66	F	mutated	none	S4A
