# Supplementary material for: In Vitro 3D Spheroid Culture System Displays Sustained T Cell-dependent CLL Proliferation and Survival
Source: Hemasphere. 2023 Aug 23;7(9):e938. doi: 10.1097/HS9.0000000000000938 (PMC10448932; doi:10.1097/HS9.0000000000000938)
Supplement: Supplementary file 3 [file hs9-7-e938-s003.pdf]

Supplemental Figure 1.

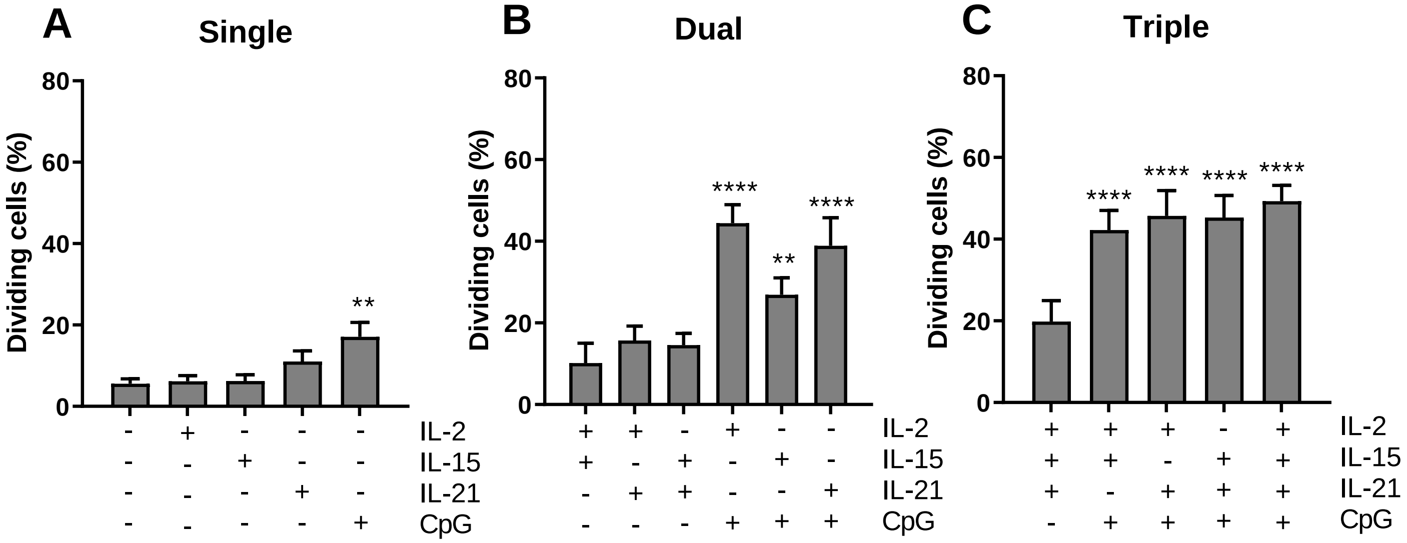

Supplemental Figure 1. CLL proliferation cocktail optimization. A-C) CFSE-labeled CLL cells were stimulated as indicated and cultured in 3D spheroid cultures for 1 week. Afterwards, cells were measured using flow cytometry and the percentage of dividing cells was quantified. **(A)** Results of single cytokine or CpG stimulation. **(B)** Results of dual cytokine stimulation or single cytokine stimulation in combination with CpG. **(C)** Results of dual and triple cytokine stimulation in combination with CpG. Bars represent the mean ± SEM (n = 9), **p<0.01, ****p<0.0001 (paired t-test). Each group was compared with the unstimulated control group.

Supplemental Figure 2.

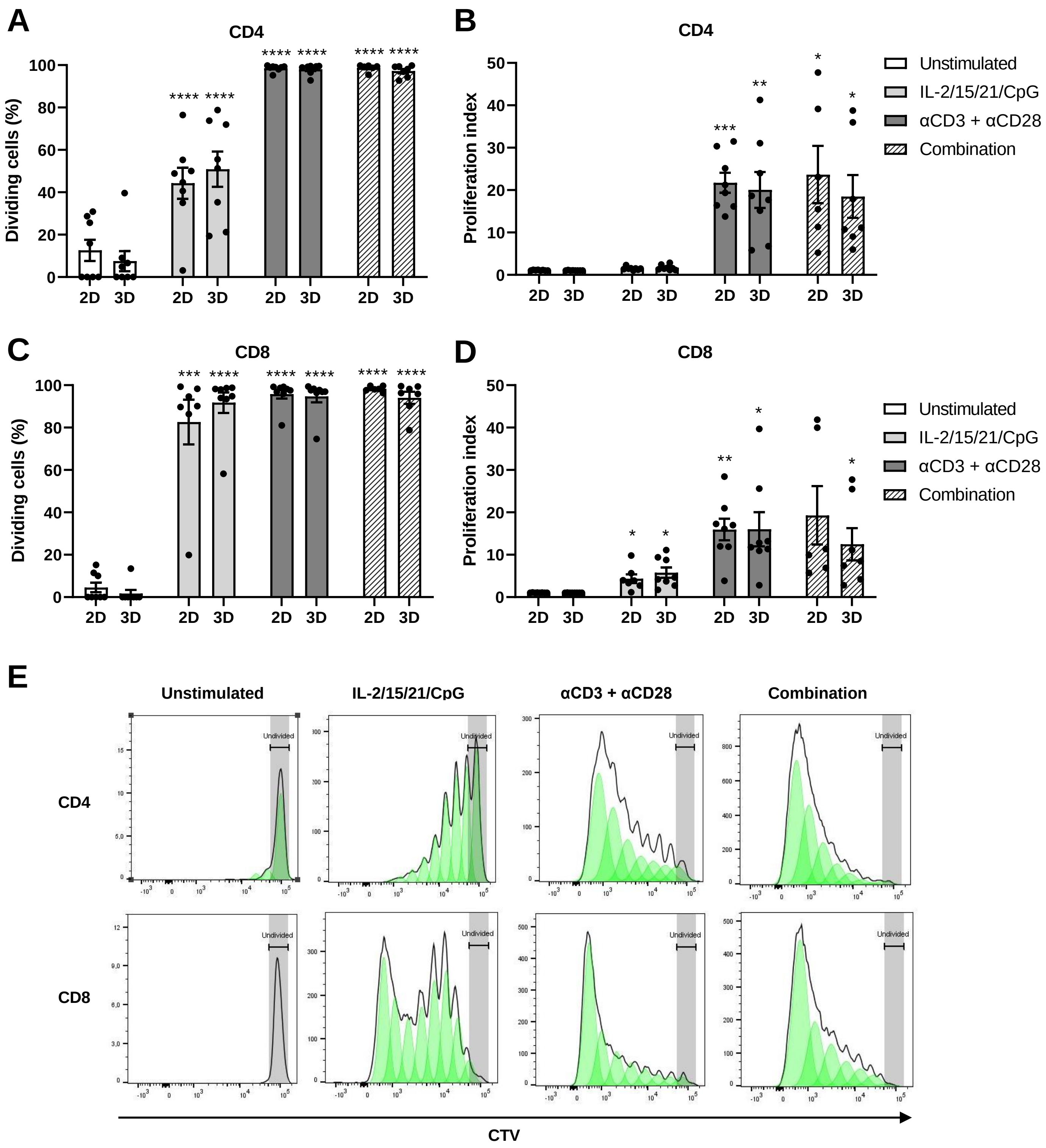

Supplemental Figure 2. Overview of T cell proliferation data. A-D) CTV-labeled CLL samples were stimulated as indicated and cultured in standard round-bottom 96 well plates (2D) or ULA plates (3D) for 8 days. Afterwards, cells were measured using flow cytometry, and both the percentage of dividing cells **(A, C)** and the proliferation index were quantified **(B, D, E)** for both CD4+ and CD8+ T cells. Bars represent the mean ± SEM (n = 10), *p<0.05, **p<0.01, ***p<0.001, ****p<0.0001 (two-way ANOVA). Each group was compared with the unstimulated control group.

Supplemental Figure 3.

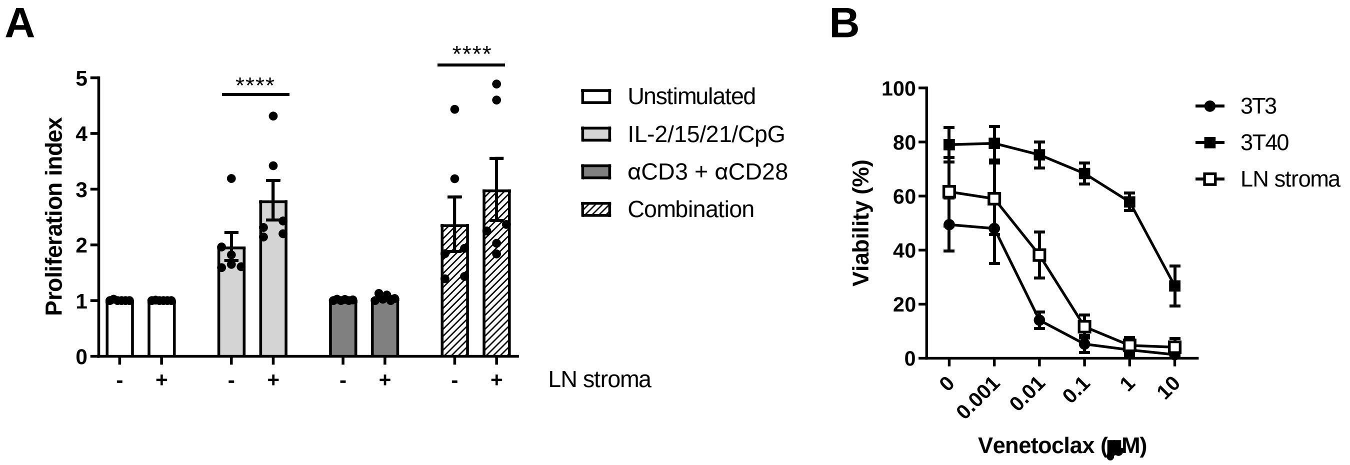

Supplemental Figure 3. Addition of lymph node stroma promotes CLL proliferation. A) CTV-labeled CLL cells were stimulated as indicated and/or cocultured with primary LN fibroblasts in 3D spheroid cultures for 1 week. Afterwards, cells were measured using flow cytometry, and the proliferation index of the CD5+CD19+ CLL population was quantified. Error bars represent the mean ± SEM (n = 6), *p<0.05 (two-way repeated measures ANOVA). **B)** CTV-labeled CLL cells were cocultured with either 3T3, 3T40 or primary LN fibroblasts in 3D spheroid cultures for 1 week. Afterwards, cells were incubated with a titration of 0.001-10µM venetoclax for an additional 24 hours. Viability was measured by flow cytometry using DiOC6 and TO-PRO-3 viability dyes. Error bars represent the mean ± SEM (n = 3).

Supplemental Figure 4.

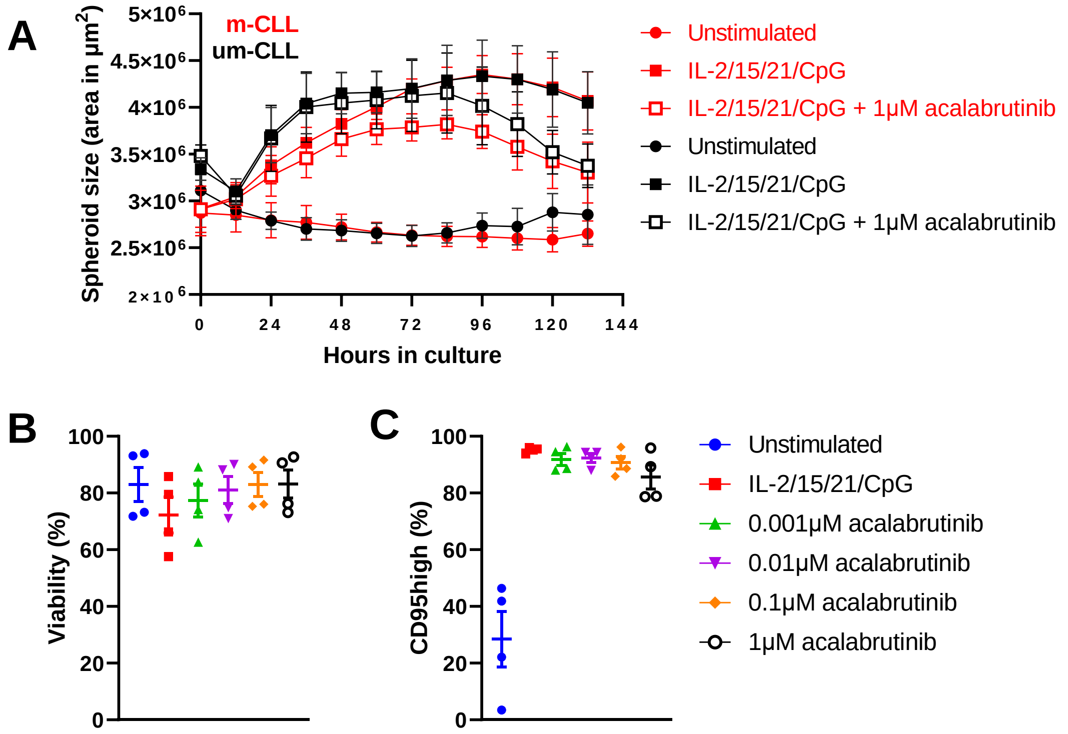

Supplemental Figure 4. CLL cell viability and activation upon in vitro acalabrutinib treatment. A) Spheroids of both unmutated IGHV (n = 5) and mutated IGHV (n = 5) CLL samples were cultured as indicated. Culture plates were placed in an IncuCyte live-cell imager which imaged and quantified the spheroid area every 12 hours. **B-C)** CTV-labeled CLL samples were stimulated with IL-2/15/21/CpG and cultured in ULA plates (3D) for 6 days in the presence of acalabrutinib. Error bars represent the mean ± SEM (n = 4). Afterwards, cells were measured using flow cytometry and the viability **(B)** and activation of the CD5+CD19+ CLL population based on CD95 expression were quantified **(C)**. These results are supplementary to the results shown in Figure 4A-B.
